# Are medical educators following General Medical Council guidelines on obesity education: if not why not?

**DOI:** 10.1186/1472-6920-13-53

**Published:** 2013-04-11

**Authors:** Anna Chisholm, Karen Mann, Sarah Peters, Jo Hart

**Affiliations:** 1 School of Psychological Sciences, University of Manchester, Lancashire, UK; 2 Division of Medical Education, Dalhousie University, Halifax, Canada; 3 Manchester Medical School, University of Manchester, Lancashire, UK

**Keywords:** Qualitative, Interviews, UK, Undergraduate education, Curriculum, Obesity

## Abstract

**Background:**

Although the United Kingdom’s (UK’s) General Medical Council (GMC) recommends that graduating medical students are competent to discuss obesity and behaviour change with patients, it is difficult to integrate this education into existing curricula, and clinicians report being unprepared to support patients needing obesity management in practice. We therefore aimed to identify factors influencing the integration of obesity management education within medical schools.

**Methods:**

Twenty-seven UK and Irish medical school educators participated in semi-structured interviews. Grounded theory principles informed data collection and analysis. Themes emerging directly from the dataset illustrated key challenges for educators and informed several suggested solutions.

**Results:**

Factors influencing obesity management education included: 1) Diverse and opportunistic learning and teaching, 2) Variable support for including obesity education within undergraduate medical programmes, and 3) Student engagement in obesity management education. Findings suggest several practical solutions to identified challenges including clarifying recommended educational agendas; improving access to content-specific guidelines; and implementing student engagement strategies.

**Conclusions:**

Students’ educational experiences differ due to diverse interpretations of GMC guidelines, educators’ perceptions of available support for, and student interest in obesity management education. Findings inform the development of potential solutions to these challenges which may be tested further empirically.

## Background

Obesity contributes to preventable death and disease and, in contrast to other lifestyle factors such as smoking, it is increasing universally [1]. Despite continuous international public health efforts to curb unhealthy lifestyle choices [2-5], obesity is now considered a global epidemic [1,6,7]. Its prominence and associations with society’s most common chronic diseases [8,9] have inevitably led to calls for doctors to take a role in helping patients manage weight as an integral part of improving and maintaining health [10-12]. The benefits of tackling obesity with patients also include the potential to reduce the associated costs and workload for health care systems. For example, estimates indicate that medical costs are 30% higher in obese individuals [13] and that one sixth of the US health care budget is spent on obesity-related illness [14].

Because harmful effects of obesity occur within almost all the body’s systems, doctors from many health care settings encounter patients who will benefit from losing weight [9]. However, research highlights that opportunities to discuss obesity with patients are missed [15,16]. The socially sensitive nature of the topic along with not knowing how to help patients lose weight can prevent these conversations from occurring [17,18]. Evidently there is an important unfulfilled role for medical education in preparing doctors in this area.

Evidence about methods to support individuals to change unhealthy behaviours exists and may address doctors’ training needs. The development of various theories outlining key behavioural determinants has resulted in the identification of a range of behaviour change techniques (BCTs) [19,20]. For example, research suggests that self-monitoring is particularly effective in eliciting increases in individuals’ fruit and vegetable intake [21]. Other examples of effective behaviour change strategies include creating implementation intentions to achieve goals [22] and motivational interviewing to reduce resistance to change [23]. Within empirical studies, patients are shown to lose weight and change dietary and activity patterns when health professionals use these kinds of behaviour change techniques [23,24]. Theories such as the PRECEDE model [25] and Social Cognitive Theory [26] can also be particularly helpful for health promotion programme designers because they highlight multi-level factors within individuals’ contexts, behaviours and environments that influence the success of behaviour change interventions. Thus a large evidence-base exists from which medical education could draw to inform curriculum developments involving obesity management.

Despite the availability of these theoretical frameworks and recommendations from the UK’s General Medical Council GMC, [27] that medical students graduate with the ability to discuss obesity and psychological aspects of behaviour change with patients, the extent to which students receive this education is unknown. Some research suggests however, that behaviour change education tends to be sporadic and presented separately from clinical experiences [28]. Surveys indicate that physical activity and smoking education are particularly poorly integrated within undergraduate medical programmes [29,30]. However, it is difficult to clearly identify behaviour change education through descriptive curriculum surveys [28] and more in-depth methods may be required to understand the nature of this education within medical school curricula. In relation to obesity, a recent systematic review identified few educational interventions for medical students (none of which were from the UK or Ireland), illustrating that it remains unclear whether medical students are receiving training in this area [31]. Due to insufficient empirical evaluations it also remains unknown what effective obesity management education entails [31].

It is possible that current medical programmes have integrated effective education in this topic already. However, reported slow uptake and poor integration of other behavioural and social science topics suggest otherwise [32,33]. Barriers such as an inability to identify appropriately qualified teaching staff and not formally assessing these topics have prevented sufficient integration within medical education [33]. However, investigations have not focused upon the barriers to providing students with education that specifically focuses on supporting obese patients to lose weight through changing unhealthy behaviours (referred to from here on as obesity management education; OME). This study explored the following research question: What are medical educators’ perceptions of the main factors which influence the inclusion and delivery of obesity education within undergraduate medical programmes?

## Methods

### Design

We conducted a qualitative study to explore the research question. Rather than administering surveys to gather educational descriptions as similar studies have done [29,30], we used semi-structured interviews to elicit participants’ views and experiences on the topic. Interviews allowed for unanticipated ideas to be pursued with participants during data collection so that factors underlying OME implementation and delivery could be explored inductively within the dataset. This study was approved by the University of Manchester Research Ethics Committee 5 (22/12/10).

### Participants

A purposive sample of educators currently involved in implementing and/or delivering OME within medical schools was sought to participate in the study. Individuals potentially meeting these criteria were identified by one researcher (AC) using information available on university websites. Thirty-four medical schools from the UK and Ireland were invited via email through directors of studies and senior staff. We asked individuals (following responses to initial emails) to nominate other educators within their school to participate if they felt they personally did not meet inclusion criteria, so that educators with the most relevant experiences could be recruited. Subsequently, 46 individuals from the 34 medical schools were invited to participate. In line with qualitative methodological principles [34], this approach also enabled the generation of a varied sample of individuals with different characteristics which increased opportunities to elicit a full range of existing views on the research topic.

### Data collection

One author (AC) conducted semi-structured telephone interviews with participants (mean duration = 29 minutes, range = 15–44 minutes). AC initially defined the term ‘obesity management’; clarifying that the focus of the interview was on lifestyle management rather than surgical or pharmacological interventions. A topic guide directed questioning around participants’ views and experiences of OME (e.g. satisfaction with quality of education; barriers and facilitators to provision of education). Participants provided written consent and interviews were digitally audio-recorded and transcribed verbatim.

### Data analysis

Data were analysed using grounded theory principles [35]. AC initially created an analysis document outlining patterns in the data which were then grouped into potential themes and subthemes. The research team (AC, SP, JH, KM) met on several occasions to discuss how closely super- and subordinate themes related to the data. Five iterations of analysis were conducted and each time the topic guide was amended so that emerging themes could be explored with participants in subsequent interviews. Analysis and data collection ceased when no new ideas arose from interviews and the identified themes and subthemes remained stable despite gathering data from new participants.

## Results

Of the 46 individuals invited, 27 from 23 different medical schools participated. Participants’ mean age was 51 years (range 29–65 years), 14 (51.85%) were female, and 24 (88.89%) were British. Participants’ educational roles and specialties/disciplines, and characteristics of medical schools included in the sample are displayed in Tables 1 and 2 (respectively).

**Table 1:** Interview study participants’ (n = 27) roles within UK and Irish medical schools and their occupational specialties/disciplines

	**Frequency of participants (%)**
Educational role within medical school*	
Delivers education [D]	6 (22.22)
Co-ordinates module/strand [C]	11 (40.74)
Leads undergraduate programme [L]	10 (37.04)
Clinical or academic specialty/discipline	
Clinical (including Rheumatology, Podiatry, Anaesthesiology, Midwifery)	5 (18.52)
General Practice (General Practitioners)	8 (29.63)
Behavioural Sciences and Education (Cognitive/Clinical/Health Psychology, Medical Education)	6 (22.22)
Public Health (Dietician, Epidemiology, Public Health Medicine/Research)	3 (11.11)
Biomedical Sciences (Biochemistry, Pharmacology, Immunology)	5 (18.52)

*D = Educators who deliver a distinct component of the curriculum that relates explicitly to obesity (and who do not have any broader roles within the medical school).

C = Educators who co-ordinate relevant modules or strands in the medical programme (may therefore deliver as well but main role to coordinate a relevant section of the curriculum).

L = Educators with a broad overview of the curriculum (may deliver distinct components as well) e.g. deans, course developers, directors of studies.

**Table 2:** Characteristics of UK and Irish medical schools (n = 23) included within the interview sample

**Characteristic of medical school**	**Number of medical schools (%)**
Century established	
1400–1899	11 (47.83)
1900–1999	7 (30.43)
2000-present	5 (21.74)
Entry level for course	
School leavers only	8 (34.78)
Graduates only	1 (4.35)
Both school leavers and graduates	14 (60.87)
Intake per year	
1–150	7 (30.43)
151–300	8 (34.78)
301-450	8 (34.78)
Medical school course description*	
Predominantly didactic	10 (43.49)
Predominantly PBL	5 (21.74)
Hybrid PBL/didactic	8 (34.78)

* Classifications of Medical school curriculum type were derived from the most recently published GMC QUABME documents (http://www.gmc-uk.org/education/medical_school_reports_full_list.asp) or where this information was not available, course description on University websites were used.

**Figure 1:**
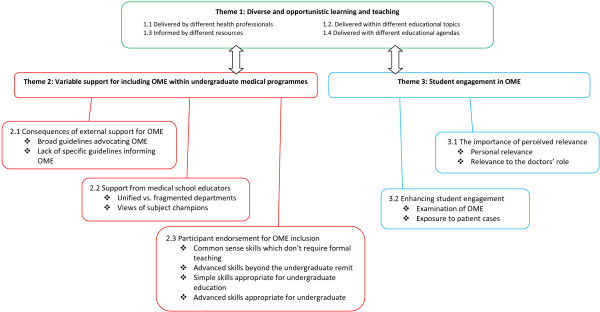
Themes and subthemes explaining the challenges of implementing and delivering OME and suggested solutions.

Three themes emerged from the data that explained which factors influenced OME implementation and delivery within medical schools: 1) Diverse and opportunistic learning and teaching, 2) Variable support for including OME within undergraduate medical programmes and, 3) Student engagement in the topic (Figure 1). Quotes from interviews are italicised and non-identifying participant codes provided in parentheses with references to individuals’ educational roles (e.g. Pt1: D). Table 1 displays definitions of educator role labels.

### Theme 1: Diverse and opportunistic learning and teaching

Participants described a diverse range of educational approaches to providing OME (Table 3). Firstly, various health professionals were reported to deliver the education including doctors, other health professionals and public health professionals/researchers. Secondly, participants highlighted that OME could *‘could fit anywhere’ (Pt7: L)* within medical programmes and reported a range of topics in which it occurred. These factors were believed to lead to variability in students’ experiences of OME. The opportunistic nature of clinical placements in particular was thought to account for the omission of this education for some students.

‘We would expect a number of obese people to come into consultations and for the GPs to opportunistically teach on the subject, I couldn’t put my hand on my heart and say yes every student gets taught about obesity’ (Pt22: C)

**Table 3:** Participants’ (n = 27) descriptions of how obesity management education is provided to students within medical schools

**Delivered by different health professionals**	**Delivered within different educational topics**	**Informed by different types of resources**	**Delivered with different educational agendas**
			To raise awareness of:
1. Audiologist	1. Adherence behaviour	1. Behavioural and social sciences literature and education network guidelines	1. Consequences of unhealthy behaviours
2. Bariatric surgery researcher	2. Central nervous system	2. Charitable organisation resources (national forums for health/obesity)	2. Current practice and team work
3. Biochemist	3. Chronic disease	3. Government guidelines (Department of Health reports/handbooks)	3. Determinants of obesity
4. Biomedical scientist	4. Clinical placements	4. Health care system guidelines (NICE/SIGN)	4. Difficulties of achieving behaviour change
5. Children’s health advocacy organisation worker	5. Communication skills	5. Personal experience (from clinical practice)	5. Effective behaviour change techniques
6. Clinicians (various specialties)	6. Endocrinology		6. Epidemiology of obesity
7. Communication skills specialist	7. Gastroenterology		7. Importance of biopsychosocial approach
8. Dietician	8. Human diversity		8. Health promotion approaches
9. Psychologist	9. Lifestyle		9. Public health issues related to obesity
10. Midwife	10. Metabolism		Skills acquisition:
11. Nurse	11. Nutrition		10. Address patients’ beliefs/barriers to change
12. Nutritionist	12. Obesity week		11. Assess patients’ self-efficacy
13. Pharmacist	13. Patient safety		12. Constructive advice regarding weight loss
14. Physiotherapist	14. Professionalism		13. Learn/use behaviour change skills
15. Public health professional	15. Psychiatry		14. Practical management of obesity with patients
15. Social worker	16. Psychology		
16. Speech and language therapist	17. Rheumatology		
	18. Surgery		

Note. The content of each column represents concepts derived directly from participants’ expressions within the dataset.

Participants also reported using different resources to inform OME content and working towards contrasting educational agendas (see Table 3). Most strikingly, whilst some OME aimed to raise student awareness about issues related to obesity, others provided students with skills to support patients to change obesity-related behaviours.

‘You said you were quite interested in lifestyle management stuff and I don’t know that we do go very far down that road really so it’s more kind of flagging up obesity as an issue and showing why it’s important’ (Pt8: C)

‘If we’re teaching them that it is an important thing to do it can be sending quite a dissonant message if then we said it’s important but don’t worry about it - learn it one day..we expect our 5th year students who do a…placement in general practice to actually achieve some behavioural change with patients’ (Pt16: L)

### Theme 2: Variable support for including OME within undergraduate medical programmes

#### Consequences of current external support for OME

Participants reported the positive influence of broad curriculum guidelines, particularly Tomorrow’s Doctors [TDs]^23^ in endorsing the inclusion of OME within curricula.

*‘It* [TDs] *was kind of fuel to the fire of, I think we need to do something and we need to be to be highlighting this but it certainly contributed, it provided support for me saying to my colleagues I think we need this in’ (Pt15: C)*

Although educators did not believe that TDs ‘*should be too prescriptive’ (Pt7: L)* they reported a lack of supplementary guidance for developing education in this area. This was compounded by the fact that educators felt they themselves lacked understanding about what methods effectively support obese patients to change unhealthy behaviours. This was thought to explain why there was more education on other lifestyle behaviours perceived to be easier topics to approach.

‘Obesity is probably fairly lightly touched upon amongst other issues like smoking…we still struggle as clinicians in our conversations with obese patients. Even just raising the topic is more difficult than smoking. If me on my clinical days I struggle with it in the practice down the road, then it’s not surprising if perhaps I struggle how to teach it to medical students’ (Pt22: C)

#### Support from medical school educators

Support internal to medical schools varied: some participants described *unified* medical school departments where support for OME was unequivocal, whereas others described *fragmented* departments in which there was disagreement among educators about how important OME was and in what discipline it fit.

*‘I don’t know that necessarily everybody thinks it’s terribly important…people tend to say “oh behavioural and social sciences” you know, and kind of wave their hand over there somewhere;* [we] *tend to be seen as a little cluster somewhere over there and slightly interchangeable’ (Pt8: C)*

Participants reported achieving successful implementation of OME due to the influence of key figures who could advocate for medical education and influence its implementation: *‘you need a few champions to take it on board’ (Pt6: D).* Participants were therefore disappointed if potential advocates missed opportunities to aid implementation.

‘I am disappointed that deans haven’t done more and including head of school…it’s a big gap in our teaching…They’ve been supportive in terms of words but they haven’t really put any pressure on the students to attend or any direct encouragement…they are giving a clear message about aspects of the curriculum which they think are vital’ (Pt27: C)

In contrast however, some participants described subject champions as individuals who inappropriately push topics into the curriculum due to personal interest.

‘It almost became a crusade for various people…it is certainly made into a priority by various people who are in the system; not everything is based on identifiable patient needs, our medical student needs, some of it is driven by professionals with an interest in that field’ (Pt2: C)

#### Participant endorsement

Participants supported the inclusion of OME within medical programmes for two reasons; 1) obesity contributes to prominent chronic diseases, and 2) doctors’ responsibilities include managing obesity with patients. Despite this, participants displayed contradictory perceptions around how advanced OM skills are in practice. Some believed obesity management is ‘common-sense’, which doesn’t require training.

‘Not everything needs to be taught in a direct [way], a lot of these things are common sense … if they know the basics of biochemistry, if they know the basics of human nutrition, the basics of human physiology, they know the basic medicine surgery that kind of stuff this kind of issue, they should be able to handle it very effectively’ (Pt2: C)

In contrast, others argued that OME involves advanced skills beyond the scope of undergraduate education.

‘They’re [medical students] not yet ready to be practicing these things…I think further down the line when they start to specialise’ (Pt18: D)

Some participants believed OM involves skills that can and should be included within undergraduate medical programmes. Within this group of participants though, disagreement existed regarding whether OM skills were basic or difficult for undergraduate students to master.

‘We’re stopping short if we don’t teach about those sorts of basic approaches to behavioural change and they are very basic so it’s not, we’re not teaching complicated processes but we’re teaching basic approaches like motivational interviewing’ (Pt16: L)

### Theme 3: Student engagement in the topic

Whilst some educators reported that students were very engaged in learning about OM, others found it difficult to elicit student interest in the topic.

#### The importance of perceived relevance

The extent to which the issue of obesity was relevant to students was reported to affect engagement levels. Firstly, the personal relevance of obesity to students was believed to help or hinder student engagement in OME.

‘There needs to be a way to make them interested…they’re all thin because they do lots of sports and they can’t relate it in their personal lives’ (Pt6: D)

‘I have had individual students who are really enthusiastic about obesity and obesity management, interestingly some of whom have obesity problems themselves’ (Pt1: D)

Secondly, unlike study participants themselves (as illustrated above), it was reported that students often did not perceive OM as being within the doctor’s role and were therefore more interested in learning about biomedical aspects of medicine than topics related to obesity management.

‘They believe it’s just somebody else’s role, their role is more sort of dealing with organic damage or more obvious manifestations of disease and illness rather than dealing with consequences’ (Pt1: D)

#### Enhancing student engagement

Because of the perceived relationship between level of student engagement in OME and how relevant they consider the topic, educators attempted to enhance student engagement by actively highlighting its relevance within educational sessions. In particular, patient cases (real or simulated) that explicitly involved obesity were used to achieve this. An additional strategy to enhance student engagement was implementing formal assessments as this communicated that OME was a priority within their undergraduate programmes.

‘That’s absolutely crucial they’ve got to think two things either oh god are they ever going to need this as a doctor or b) will they be examined on it. So we also put exam questions in and we make that clear’ (Pt6: D)

### Implications of the themes to address educators' challenges

We suggest a number of practical solutions to the challenges highlighted by the study findings (see Table 4). Firstly, in order to guide educators in selecting educational approaches which coincide with curriculum recommendations [27] and reduce variability in student experience, we suggest that a statement detailing the core objectives of OME is produced to complement the GMC’s competency requirements for including it within undergraduate programmes. It may be beneficial for the statement to specify particular competencies students are expected to accomplish and identify key components of consultations involving obesity management. For example, ‘students will demonstrate the ability to 1) raise the topic of obesity management with patients 2) include effective behaviour change techniques within discussions of obesity management with patients 3) refer patients to appropriate services and resources.’ This level of specificity could assist educators in ensuring all students are exposed to key elements of OME and help them meet GMC recommendations without placing inflexible restrictions upon how it is delivered.

**Table 4:** Problems associated with OME based upon interview study findings and suggested solutions

**Theme**	**The problem**	**Suggested solution**
1. Diverse and opportunistic learning and teaching of obesity management education (OME)	The type and extent of OME delivered to medical students varies widely, indicating that GMC recommendations are interpreted differently and that training for future doctors is inconsistent.	Dissemination of a clear statement detailing broad educational objectives in relation to OME. For example, ‘Students will demonstrate the ability to 1) raise the topic of obesity management with patients 2) include effective behaviour change techniques within discussions of obesity management with patients 3) refer patients to appropriate services and resources.’
2. Existing support for including OME within undergraduate medical programmes	External guidance for educators designing OME is lacking and there is mixed support for the inclusion of OME within medical schools.	Increase access to evidence-based, content-specific guidelines and within this, include effective behaviour change techniques to improve awareness of the skills involved in supporting patients with managing obesity and demonstrate its suitability for inclusion at the undergraduate level.
3. Student engagement in OME	Whilst some educators experience students who are interested in learning about obesity management, others encounter difficulty engaging students.	Implement recommendations to enhance student engagement in learning about obesity management through tailoring education to highlight its relevance to students as future doctors and by including real patient cases where possible and including explicit assessment on OME.

Theme 2 illustrated that educators felt unsupported in selecting optimal educational content for OME. We therefore propose that educators are provided with content-specific guidelines on obesity management, particularly as there is available evidence-based literature outlining behaviour change techniques suitable for use by health professionals [19,36]. This should improve educators’ awareness of the skills involved in supporting patients with managing obesity and thus provide better support for them in selecting content for medical programmes. In addition, this could also address issues identified within theme 2 regarding confusion around the complexity of behaviour change skills and therefore how suited they are to being included at the undergraduate level. By making behaviour change skills more transparent to educators, and demonstrating that they can be implemented within clinical interactions [23,24], conflicting perceptions between educators about the level of difficulty involved in learning obesity management skills may be reduced.

Finally, theme 3 illustrated diversity in educators’ experiences of engaging students in learning about obesity management. Educators consistently emphasised the importance of creating education that feels relevant to students to stimulate motivated learners. Although the association between relevance and student engagement has been highlighted previously [37], it seems that a more consistent approach to designing OME that is directly relevant to medical students is needed. Based on the above findings, we propose that educators ensure OME is tailored to highlight its relevance to students both professionally and personally; for example by including real patient cases (to demonstrate its relevance to the doctor’s role) and explicit assessments within medical programmes (to demonstrate its relevance to students as learners).

## Discussion

This study demonstrates that inconsistency within UK and Irish OME derives from a lack of clarity and consensus about how to design and deliver this education. Previous research has identified the challenges of integrating comparable lifestyle-related topics such as smoking and physical activity within medical programmes [29,30,33]. Thus this study suggests that barriers to curricula integration remain, even for high priority, topical issues like obesity [7]. We therefore offered some practical suggestions for moving forward. We have also drawn from a broad sample of medical educators in order to better understand these issues including the lack of clarity regarding who is best placed to deliver OME and where it should be located within medical programmes. A key finding within our study was that educators believed that the opportunistic and multi-disciplinary nature of obesity management largely accounted for inconsistent student experiences. A notable consequence of this is that some students may receive no formal education in this area at all, suggesting a failure to meet GMC recommendations that all medical graduates should be able to discuss obesity and behaviour change with patients (GMC, 2009).

Another key finding reported by educators was that OME can be delivered within numerous areas of the curriculum. Although this may assist with integrating OME within existing programmes without adding to the pressures of already overloaded curricula [38,39], it may also reflect a lack of understanding about how to deliver optimal OME. Although this is understandable given the lack of available evidence on this [31], guidelines on designing and integrating medical education in this domain have recently been developed and may be helpful for educators [37]. Our findings also identified disparity between the reported educational agendas that guided OME objectives; some focused upon raising student awareness about obesity whereas others aimed to equip students with weight management skills. This suggests that GMC recommendations on this topic (GMC, 2009) have been interpreted differently and that competency levels expected of students in this area may vary considerably across medical schools. Thus it may be beneficial for future recommendations to specify some common OME objectives to clarify the competencies medical students are expected to achieve (Table 4).

Participants’ views of current resources for developing OME revealed a tension between the useful influence of curriculum guidelines (GMC, 2009) in advocating its inclusion within curricula, and the lack of supplementary guidance to inform educational content. Although educators wanted specific guidance on how to teach OME, there is a paucity of evidence to inform these educational decisions [31]. There is however, a large evidence-base which has defined theory-informed behaviour change techniques [19,20]; which have produced some desirable changes to health behaviours and health outcomes [21,23,40,41]. Better application of this literature to medical education is therefore required and could address this challenge for educators.

We identified conflicting accounts regarding support within medical schools. Whilst some viewed educators involved in providing OME as valuable subject champions, others believed they created unhelpful interest groups. Participants expressed other contradictory views about how complex OM skills are and how appropriate it therefore is to provide this education to medical students. We could not determine the impact of these contrasting views upon student experiences; however, the role of the hidden curriculum (i.e. educators’ implicit views) in preventing successful curriculum reform has been identified previously [42,43]. Research in this area suggests that implicit beliefs and attitudes of educators can influence students’ learning and future career choices [44]. Thus it may be that the views of educators reported in our study affect the provision of OME within medical schools and the likelihood of students addressing this issue with future patients. We therefore advocate the dissemination of content-specific guidelines within medical schools which would alert educators to the evidence-base for effective behaviour change techniques and demonstrate that skills teaching in this area is suitable at the undergraduate level. This may in turn promote more consistent support for its inclusion within undergraduate programmes.

Although the GMC advocates including OME within medical programmes and their recommendations are highly valued by medical schools [45], evidence also shows that due to various barriers, the implementation of topics related to public health has been slower than others [32,33]. One factor suggested to influence the slow uptake of health promotion education is poor student engagement [32]. We heard contrasting accounts of how engaged students were in OME, but participants agreed that enhancing relevance to students was key to improving engagement in the topic. Confusion about doctors’ roles in encouraging lifestyle change in patients is continuously reported by clinicians [17,46], suggesting that clarification on this issue by health care governing bodies is needed to resolve some of the issues raised in this study. Additionally, participants in our study and educators in others [47] have indicated that exposing students to experiences in clinical settings can help in improving student engagement in the topic and in clarifying the doctors’ role in OM.

Finally, along with these recommendations for individuals involved in implementing OME, it is also important to recognise the role of wider contextual issues. For example, in order to support the successful translation of the above recommendations, attention should also be given to cultivating a supportive environment within medical schools. In line with research highlighting that unsupportive environments can prevent effective education delivery [33], it may be beneficial to also consider institutional level interventions which address educators’ views and attitudes across medical schools towards changing aspects of curricula and including topics such as OME. Taken together, these findings highlight some uncertainty regarding how the medical education is designed and developed. For example, it is unclear how pressing public health issues such as obesity should influence curriculum content; whether educators should be reactive or pro-active about this; and also who is responsible for making these decisions about the evolving nature of medical curricula. Although the present study has not investigated or addressed these issues, it does expose key ambiguities around this topic. Resolving some of this ambiguity may in turn support the production of more consistent and pragmatic education for students.

As obesity is relevant to many areas of medicine, and therefore medical education, it is likely that we did not elicit accounts from individuals across all contexts involving OME. Comparable education in communication skills and behavioural and social sciences have also encountered problems in identifying what, where and how this education is delivered within medical programmes [33,48]. This is supported by participants’ reports that it was difficult to accurately locate OME within medical programmes and that they may have been unaware of other educators who deliver OME elsewhere in the programme. Although the range of educators in our sample (Table 1) suggest that the findings draw upon a variety of contexts, it is possible that our results were influenced by having larger proportions of general practitioners and psychologists than other health professionals within the sample. It was also not possible to obtain views from educators delivering informal education to students within programmes, despite participants’ reporting that this likely made up a substantial proportion of teaching on OME. These limitations themselves support the finding that OME is inconsistently delivered within medical programmes.

A further limitation is that the range of views elicited may have been restricted by recruiting individuals who support OME within medical programmes. Additional barriers may exist for educators with more negative views about OME and we might expect such views to indicate personal barriers (e.g. attitudes about OME) rather than some of the external barriers indentified within our study (e.g. lack of resources). However the potential for bias in this way was reduced by the inclusion of accounts from educators with different opinions about the extent to which OME should be included within medical schools. Finally, although this study allowed authors to suggest means of addressing the challenges identified within the findings, further research is needed to explore the feasibility and efficacy of these potential solutions.

## Conclusions

This study explains the discordance between recommendations by governing bodies to develop doctors who are proficient in supporting patients to change unhealthy behaviours GMC, [10,27,49,50] and parallel evidence indicating that doctors feel underprepared by medical education to do this [17,18]. The current findings highlight that the challenges associated with integrating OME and remain unresolved within UK and Irish medical schools. Potential areas of intervention to address this include: reducing uncertainty around what optimal methods of providing OME involve through defining core educational objectives; improving external and internal levels of support for OME via dissemination of evidence-based context-specific guidelines to educators; and improving engagement by enhancing the relevance of OME to students.

## Abbreviations

UK: United Kingdom; GMC: General Medical Council; OME: Obesity Management Education; TD: Tomorrow’s Doctors; OM: Obesity management.

## Competing interests

The authors declare that they have no competing interests.

## Authors’ contributions

All authors conceived the study and contributed to its design. Recruitment was facilitated by all authors and operationalised by AC. AC carried out the data collection and initial analysis. JH, SP, and KM contributed to further analysis. All authors contributed to writing the manuscript and commented on multiple drafts. All authors read and approved the final manuscript.

## Authors’ information

AC is a PhD student at the School of Psychological Sciences, Faculty of Medical and Human Sciences, University of Manchester UK. KM is Professor Emeritus in the Division of Medical Education at Dalhousie University in Halifax, Canada, and Professor and Part-time Chair in Medical Education, Manchester Medical School in Manchester, United Kingdom. SP is Senior Lecturer at the School of Psychological Sciences, Faculty of Medical and Human Sciences, University of Manchester UK. JH is a senior lecturer in communication at Manchester Medical School, University of Manchester.

## Pre-publication history

The pre-publication history for this paper can be accessed here:

http://www.biomedcentral.com/1472-6920/13/53/prepub
